# Dissolution and Hydrolysis of Bleached Kraft Pulp Using Ionic Liquids

**DOI:** 10.3390/polym11040673

**Published:** 2019-04-12

**Authors:** Guillermo Reyes, María Graciela Aguayo, Arturo Fernández Pérez, Timo Pääkkönen, William Gacitúa, Orlando J. Rojas

**Affiliations:** 1 Departamento de Ingeniería en Maderas, Facultad de Ingeniería, Universidad del Bío-Bío, Av. Collao 1202, Casilla 5-C, Concepción C.P. 4081112, Chile; maguayo@ubiobio.cl (M.G.A.); wgacitua@ubiobio.cl (W.G.); 2 Nanomateriales y Catálisis para Procesos Sustentables, Departamento de Ingeniería en Maderas, Facultad de Ingeniería, Universidad del Bío-Bío, Av. Collao 1202, Casilla 5-C, Concepción C.P. 4081112, Chile; 3 Departamento de Física, Facultad de Ciencias, Universidad del Bío-Bío, Av. Collao 1202, Casilla 5-C, Concepción C.P. 4081112, Chile; arturofe@ubiobio.cl; 4 Department of Bioproducts and Biosystems, School of Chemical Engineering, Aalto University, Espoo P.O. Box 11000, Finland; timo.paakkonen@aalto.fi; 5 Biobased Colloids and Materials, Department of Bioproducts and Biosystems, School of Chemical Engineering, Aalto University, Espoo P.O. Box 11000, Finland; orlando.rojas@aalto.fi

**Keywords:** cellulose dissolution, ionic liquid, nanocellulose, green technology

## Abstract

Forestry industries in Chile are facing an important challenge—diversifying their products using green technologies. In this study, the potential use of Ionic Liquids (ILs) to dissolve and hydrolyze eucalyptus wood (mix of *Eucalyptus* *nitens* and *Eucalyptus* *globulus*) kraft pulp was studied. The Bleached Hardwood Kraft Pulp (BHKP) from a Chilean pulp mill was used together with five different ILs: 1-butyl-3-methylimidazolium chloride [bmim][Cl], 1-butyl-3-methylimidazolium acetate [bmim][Ac], 1-butyl-3-methylimidazolium hydrogen sulfate [bmim][HSO_4_], 1-ethyl-3-methylimidazolium chloride [emim][Cl], 1-ethyl-3-methylimidazolium acetate [emim][Ac]. Experimentally, one vacuum reactor was designed to study the dissolution/hydrolysis process for each ILs; particularly, the cellulose dissolution process using [bmim][Cl] was studied proposing one molecular dynamic model. Experimental characterization using Atomic Force Microscopy, conductometric titration, among other techniques suggest that all ILs are capable of cellulose dissolution at different levels; in some cases, the dissolution evolved to partial hydrolysis appearing cellulose nanocrystals (CNC) in the form of spherical aggregates with a diameter of 40–120 nm. Molecular dynamics simulations showed that the [bmim][Cl] anions tend to interact actively with cellulose sites and water molecules in the dissolution process. The results showed the potential of some ILs to dissolve/hydrolyze the cellulose from Chilean Eucalyptus, maintaining reactive forms.

## 1. Introduction

The Chilean forestry industries face challenges and opportunities mainly due to the growth of competitive global industries producing a wide variety of high-value products based on forest and pulp recycled materials [1,2]. In this scenario, the Chilean forestry companies are shifting their traditional pulp mills to more diverse processes to produce higher-value products from lignocellulose raw or recycled materials, ensuring that their productivity and competitiveness is at the level of the world market [1,3].

Cellulose is the most abundant organic polymer in nature; besides being renewable, this material has shown various attractive properties, such as biocompatibility, biodegradability, mechanical and thermochemical stability [4,5]. The cellulose is mainly obtained from wood in fiber form (mixed with hemicellulose and lignin) as is one of the main constituents of the primary plant cell wall [6]. This fiber is intended primarily for the paper industry, fiber processing textiles, plastics, cosmetics, and pharmaceuticals, among others [5,6,7]. Particular attention is currently focused on clean cellulose and wood fiber synthesis because these are considered a virtually inexhaustible source of bioproducts and biofuels [5]. Cellulose is a linear polysaccharide consisting of chains of glucose monomers linked through covalent β-(1->4) bonds. These polymer chains are linked together by intermolecular and intramolecular hydrogen bonds, thereby hindering the cellulose separation process [6].

Aqueous and traditional organic solvents such as sulphuric acid (H_2_SO_4_), *N*-Methylmorpholine *N*-Oxide (NMMO), and alkali/additive solvent systems such as sodium or lithium hydroxides between specific concentrations range with the addition of urea or thiourea [8,9,10], have been used in the cellulose and pulp industries, with issues such as high cost, challenging recovery, low selectivity, low solvation, high energy consumption related (pressure, temperature and time), generation of toxic and poisonous agents; the last factor being an important issue from the point of view of sustainability [5,6]. 

Currently, some ionic liquids (ILs) are used as green solvents to dissolve and derivatize cellulose [11,12]. ILs have shown to be tunable reagents to partially or entirely remove environmental impacts [13,14,15]. Cellulose can be dissolved in ILs directly without previous treatment and might be regenerated into various forms such as fibers, films, beads or other types, by the addition of water or others non-solvents [11]. Many ILs have proven to dissolve cellulose successfully; among these, IL 1-butyl-3-methylimidazolium chloride ([bmim][Cl]) is one of the most widely used [16]. Some ILs have shown a considerable degree of toxicity; hydrophobic and long side chain substituted ILs can destroy the cell membrane and act as neurotoxins [17]. Therefore, the presence of any residual IL should be avoided in the final products. However, initial toxicity results suggest that there are no major toxicity issues related to the typical ILs used for cellulose dissolution [18,19,20]. ILs are currently used in cellulose products’ technologies, paying attention to ILs recovery/recyclability, which in some cases might be performed by distillation [21,22,23]. On the other hand, previous works have reported CNC synthesis by ILs. Abushammala et al. [24] reported the synthesis of CNC with an aspect ratio of 65 using [emim][Ac]; Iskak et al. [25] reported nanocrystals of 9 nm size using [bmim][Cl], and Man et al. [26] reported CNC using [bmim][HSO_4_] under FESEM examination with lengths of 50–300 nm and diameters around 14–22 nm.

In this work, experiments were conducted to dissolve and hydrolyze Chilean Eucalyptus wood (Bleached Hardwood Kraft Pulp (BHKP)) in ILs. Scanning Electron Microscopy (SEM), Atomic Force Microscopy (AFM), zeta potential (Φ_z_) and charge (ζ) measurements were used to characterize the morphology and stability of such fibers.

On the other hand, to improve the cost-effectiveness ratio in the ILs + cellulose processes, it is necessary to get a deeper phenomenological understanding of the physical systems involved. In this respect, recent studies have used Molecular Dynamics (MD) to elucidate the specific interactions mechanism responsible for the cellulose dissolution [27,28,29]. These studies indicated that cellulose has a high tendency to establish hydrogen bonds with ILs’ anions, therefore significantly disrupting the intrachain and interchain cellulose hydrogen bonds. Although these researches have been able to adequately describe the molecular systems and their specific interactions through All Atoms (AA) and United Atoms (UA) force fields, it is necessary to implement a Coarse-Grained (CG) force field for these complex systems due to its sluggish dynamics behavior. The CG force field will allow larger scale (size and time) simulation systems with a minor computational cost and a reasonable loss of specific system details [30,31,32]. In this work, a CG-MD model has been proposed to perform simulations of bulk properties for the mixture of cellulose + H_2_O + IL(1-butyl-3-methylimidazolium chloride). This MD model, together with the dissolution/hydrolysis process (unoptimized), serves as the starting point to explore the IL potential to dissolve/hydrolyze Chilean Eucalyptus cellulose pulp, and at the same time establish the scientific base to comprehend the elemental forces involved in the IL cellulose dissolution process.

## 2. Materials and Methods 

### 2.1. Materials

The Eucalyptus Bleached Hardwood Kraft Pulp (BHKP) provided by one Chilean Kraft Pulp Mill was used in all the experiments. The chemical composition of the BHKP is listed in Table 1. 

Chemical reagents: dimethyl sulfoxide or DMSO (CAS No.67-68-5, purity ≥ 99.7), 1-butyl-3-methylimidazolium chloride or [bmim][Cl] (CAS No.79917-90-1, purity > 98%), 1-butyl-3-methylimidazolium acetate or [bmim][Ac] (CAS No.284049-75-8, purity > 95%), 1-butyl-3-methylimidazolium hydrogen sulfate or [bmim][HSO_4_] (CAS No. 262297-13-2, purity > 95%), 1-Ethyl-3-methylimidazolium chloride or [emim][Cl] (CAS No.65039-09-0, purity = 98%) and 1-Ethyl-3-methylimidazolium acetate or [emim][Ac] (CAS No. 143314-17-4, purity = 97%) were purchased from Sigma-Aldrich and stored in inert atmosphere until its use. The ILs’ water content before drying was measured for all ILs by Karl Fischer titration, as in our previous work [33]. The water content of all ILs previous drying was less than 2800 ppm or 0.28% *w*/*w*. Therefore, a vacuum drying step was added in order to ensure a water content lower than this percentage. Despite this, it was challenging to precisely measure the water content of the IL in the pulp dissolving process due to environment condensed water and that within the pulp. 

### 2.2. Experimental and Molecular Methods

#### 2.2.1. Dissolution and Hydrolysis

**Figure 1:**
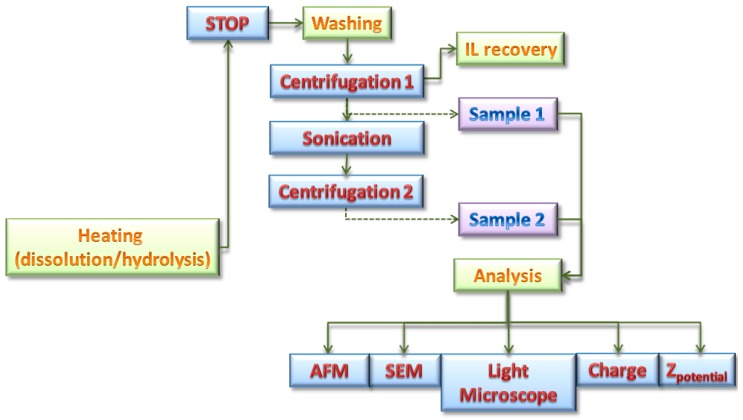
Schematic procedure for dissolution/hydrolysis of BHKP on ILs.

BHKP samples were treated with ILs: [bmim][Cl], [bmim][Ac], [bmim][HSO_4_], [emim][Cl], [emim][Ac] according to the treatment methodology presented in Figure 1.

**Figure 2:**
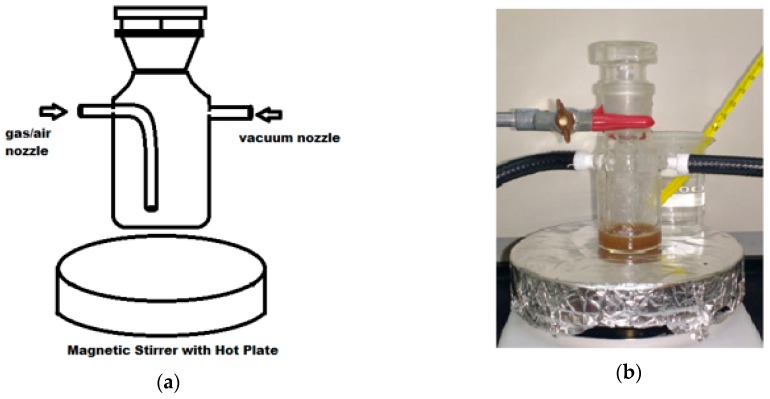
Vacuum glass reactor made for inert atmosphere dissolution and hydrolysis of BHKP using ILs: (**a**) Schematic representation of the main components; (**b**) Photograph of the laboratory reactor.

The dissolution/hydrolysis process consists of three main steps: dissolution, washing, and analysis. In the first step, a 20 g mixture was prepared containing 5% *w*/*w* BHKP and 95% *w*/*w* of IL. ILs and pulp samples were previously vacuum dried overnight (60 °C, 18 h, 0.5 bar) before mixing. However, the ILs used are highly hygroscopic, and during the dissolution process some condensed water is likely to be present. This water content and remaining acid content from the ILs synthesis can promote hydrolysis reactions [34,35]. The IL and pulp were mixed under vigorous agitation (700 rpm), followed by a 2 h dissolution/hydrolysis process at 0.5 bar vacuum pressure. The thermal treatments were carried out in a self-made glass vacuum reactor designed for this purpose (see Figure 2).

Dissolution/hydrolysis processes were carried out in the reactor at 0.5 bar of vacuum pressure (100 °C, 2 h, 700 rpm) using magnetic stirrer equipment with a hot plate. After the heating step, the physical process was stopped by the addition of cold deionized water (10 mL, Milli-Q^®^ water), promoting the pulp coagulation and solubilization of hydrolyzed fractions. 

The washing step started with centrifugation (see Figure 1) (Centrifuge Model GL21M, Changsha Yingtai Instruments Co., Changsha, China), which was carried out five times at 12000 rpm, 30 min, collecting the supernatant for ILs recovery by rotary evaporation process (rotary evaporator Hei-VAP, Schwabach, Germany); the precipitate fraction containing the treated pulp was isolated (Sample 1 in Figure 1).

#### 2.2.2. Molecular Simulation of [bmim][Cl] + Cellulose + Water

MD simulations were applied to model the behavior of systems composed of cellulose, [bmim][Cl] and water. Molecular dynamics consists of solving Newton’s classical equations of motions for a system composed of atoms (or sites). Solving such equations demands an explicit form for the potential energy *U(r*_ij_*)* for intramolecular and intermolecular interactions contributions. In the present work, the Mie Potential (equation 1) was used to compute the attractive-repulsive interactions, where *ε*_ij_ represents the depth of the potential well and *r*_oij_ the distance to the minimum potential.

(1) $$U_{vdw}\left(r_{\mathrm{ij}}\right)=U_{Mie}\left(r_{\mathrm{ij}}\right)=\frac{\varepsilon_{\mathrm{ij}}}{n_{\mathrm{ij}}-m_{\mathrm{ij}}}\cdot \left(m_{\mathrm{ij}}\cdot {\left(\frac{r_{0\mathrm{ij}}}{r_{\mathrm{ij}}}\right)}^{n_{\mathrm{ij}}}-n_{\mathrm{ij}}\cdot {\left(\frac{r_{0\mathrm{ij}}}{r_{\mathrm{ij}}}\right)}^{m_{\mathrm{ij}}}\right)$$

Parameters *n*_ij_, *m*_ij_, are the corresponding attractive and repulsive exponents, respectively. In this work, *n*_ij_ and *m*_ij_, are fixed at 6 and 9, respectively. The parameter *r*_oij_ is the intermolecular distance corresponding to the minimum interaction energy between two sites and its relation to the collision diameter σ_ij_ is given by:

(2) $$r_{0}{}_{\mathrm{ij}}=\sigma_{\mathrm{ij}}\cdot {\left(\frac{n_{\mathrm{ij}}}{m_{\mathrm{ij}}}\right)}^{\left(\frac{1}{n_{\mathrm{ij}}-m_{\mathrm{ij}}}\right)}$$

**Figure 3:**
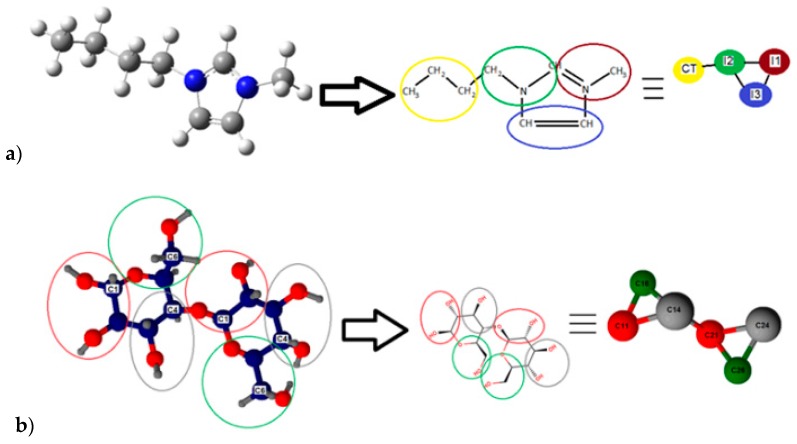
Coarse-grained models: (**a**) IL 1-butyl-3-methylimidazolium chloride ([bmim][Cl]) cation representation; (**b**) cellobiose representation.

In general, for a complete molecular description, every single atom is supposed to be modeled under force field parameters shown in Equations (1) and (2). Nevertheless, it is possible to cluster several atoms in one single site, thus reducing the computational cost with a reasonable loss of molecular detail [36]. This method called Coarse-Grained modeling [36,37,38] was adopted to model IL [bmim][Cl] and cellulose, as detailed in Figure 3. 

For IL [bmim][Cl], the five-site CG model was used and optimized based on previous works [39,40]; see Figure 3a. For cellulose, a CG force field consisting of six sites for each cellobiose unit was used; see Figure 3b. Each unit has twenty one Lennard-Jones pair contributions, seven bonding, four bending, and eight dihedrals contributions respectively, according to Bu et al. model [41]. As is shown in Figure 3a, four sites form the IL’s cation, and the chloride ion is modeled with only one additional site (see Table 2). The SPC/E traditional model with fixed angles and bonds was used for water [42]. The molecular simulation water content was selected as the maximum water content, where typically these ILs are still able to dissolve cellulose [43,44,45]. Therefore, the molecular simulation conditions were not chosen to mimic the experimental dissolution conditions but to study the dissolution phenomena in extreme water content conditions. 

Table 2 provides the van der Waals type interaction parameters. On the other hand, electrostatic contribution was given by Coulomb’s potential [46]. The large range electrostatic interaction contribution is calculated by the Ewald sum method with a convergence parameter of 0.18520 Å and *k_max1_* = 8, *k_max2_* = 8, *k_max3_* = 16 maximum values for the reciprocal lattice. NPT and NVT ensembles were implemented using the Nosé-Hover thermobarostat (f_1_ = 0.50 ps, f_2_ = 3.0 ps) and the Nosé-Hover thermostat (f = 0.5 ps), respectively. The simulations started with a lattice configuration at 100 K; then, they were quenched to the simulation temperature. DLPOLY© software [47] was used to perform all the simulations. Finally, the Lorentz-Berthelot classical mixing rules for cross-vdW interactions and 0.004 ps time step were used in all cases [46]. 

NPT simulations were carried out to predict the liquid density of pure [bmim][Cl] over the temperature range 298.15 K < T < 363.15 K. For these simulations, a Nosé-Hoover thermo-barostat was used with 0.50 ps and 3.0 ps constants, together with a cutoff radius of 15 Å [39]. The initial configuration consists of 256 molecules (1280 sites) in a lattice configuration. This lattice was equilibrated at the initial temperature of 100 K during 800 ps. At the end of this stage, the simulation started at the desired temperature. Density was calculated from average volume after the equilibrium state was observed; this was observed after 40 ns for all temperatures. The error in the density calculation was derived from the standard deviation of the volumes.

### 2.3. Instrumental Methods

#### 2.3.1. Scanning Electron Microscopy (SEM)

Sample 1 (see Figure 1) composed of solid hydrolyzed and regenerated cellulose was first seen under a light microscope (Leica, DM4B, Wetzlar, Germany) capturing images of cellulose samples at 35X. Additionally, Scanning Electron Microscopy (SEM) was used to obtain images of sample 1 at 1500X (SEM, JEOL JSM6610LV, JEOL Ltd., Tokyo, Japan).

SEM samples were prepared following four steps: (1) samples are diluted 1:10 with milli-Q^®^ water and sonicated (Elmasonic E-60H, ELMA, Singen, Germany) for 30 min. (2) Three drops of the sample are deposited on an aluminum stub previously covered with carbon tape. (3) Stubs with the drops are dried for 30 min in a vacuum oven (Medcenter Einrichtunger GmbH) at 35 °C and 0.5 bar vacuum pressure. Step (3) is repeated five times in order to obtain five drops in the same place. (4) Samples are sputtered with 100 angstroms layer of gold (DENTON VACUUM Sputter). Lastly, images from SEM are taken using an SEI detector (Secondary Electron Image) with 5kV voltage. 

After the SEM analysis, the remaining precipitate was separated in two fractions (microfibers and nanofibers) by sonication (5 min, 30% amplitude), using a digital sonifier (Branson 450, Digital Sonifier, Danbury, Connecticut, USA). The sonication process was followed by vacuum filtration (filter paper Whatman, Grade 1). The sonication-filtration processes were repeated three times until a clear suspension was obtained. 

The final washing step considers centrifugation (see Figure 1) at 12000 rpm and 10 min, after which a supernatant sample was obtained (sample 2.), which is supposed to be composed mainly of nanocellulose. This sample was analyzed by AFM, zeta potential, and charge measurements. 

#### 2.3.2. Atomic Force Microscopy (AFM)

The surface morphology of Sample 2 was examined by an AFM, Nanosurf model NaioAFM. The equipment was operated in tapping mode using PPP-FMAuD Gold Coated and a Force Modulation AFM Probes (Nanosensors) at a resonance frequency of about 75 kHz with a spring constant of 2.8 N/m. The particle sizes of the CNC samples were measured from the images using image analysis software (Image J, Fiji distribution, open-source) [48]. 

#### 2.3.3. Zeta Potential and Charge

Zeta potential (Φ_z_) was measured in samples with solid concentration of 0.05% *w*/*w* (pH = 6.0, 25 °C) using a Zeta potential equipment (Malvern Panalytical, Zetasizer Nano S, Worcestershire, UK), and charge (ζ) measurements were carried out by conductometric titration. 

Carboxyl content of the cellulosic samples was analyzed by conductometric titration according to the standard SCAN-CM 65:02 [49]. The titration was executed using an automatic titration device (Methrom 751 GPD Titrino and Tiamo 1.2.1 software, Metrohm AG., Herisau, Switzerland). The data of the titration was processed with OriginPro 2018b software (OriginLab Corporation, Northampton, MA, USA). A blank sample (water) was used to exclude systematic error within the analysis. 

## 3. Results and Discussion

### 3.1. Dissolution Process

#### 3.1.1. Experimental Results

**Figure 4:**
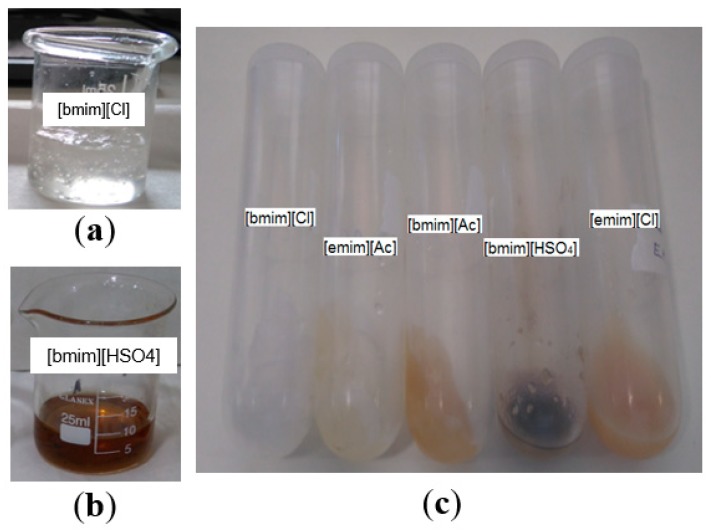
Cellulose pulp treated with ILs: (**a**) treated with [bmim][Cl] before washing; (**b**) pulp treated with [bmim][HSO_4_] before washing; (**c**) pulp samples treated with the corresponding ILs after centrifugation and IL removal.

The synthesized pulp after thermal treatment with ILs was collected and analyzed. These samples are called Sample 1 (according to Figure 1) and are supposed to be composed mainly of hydrolyzed pulp and regenerated pulp. The samples for all ILs are shown in Figure 4.

Figure 4 presents the appearances of dissolved pulp samples using ILs, before and after washing. In particular, Figure 4a,b show pulp samples dissolved with [bmim][Cl] and [bmim][HSO_4_] before washing. Figure 4a shows that the pulp treated with IL [bmim][Cl] forms a very stable viscous and transparent hydrogel. This behavior has been widely explored and attributed to the dependency of cellulose molar mass on the viscosity of the resulting mixtures with [bmim][Cl] [50], and the effect has been taken into account for the cellulose-ILs ion gel and hydrogel membranes synthesis [51,52,53]. We will discuss further that this behavior is not only due to the nature of cellulosic fibers, but also to the primary molecular interactions of the IL with water molecules that might be present in the mixture.

On the other hand, from Figure 4b, it is observed that thermal treatment with [bmim][HSO_4_] produces a solution with a darker color, thus indicating possible hydrolysis/degradation reactions taking place [54,55,56]. Even more, from Figure 4c it is possible to notice that after washing the resulting pulp samples show different colors and textures; for instance, with the ILs [bmim][Ac], [bmim][HSO_4_], and [emim][Cl], a darker pulp was regenerated in contrast to a whiter pulp regenerated in the case of [bmim][Cl] and [emim][Ac] ILs. 

**Figure 5:**
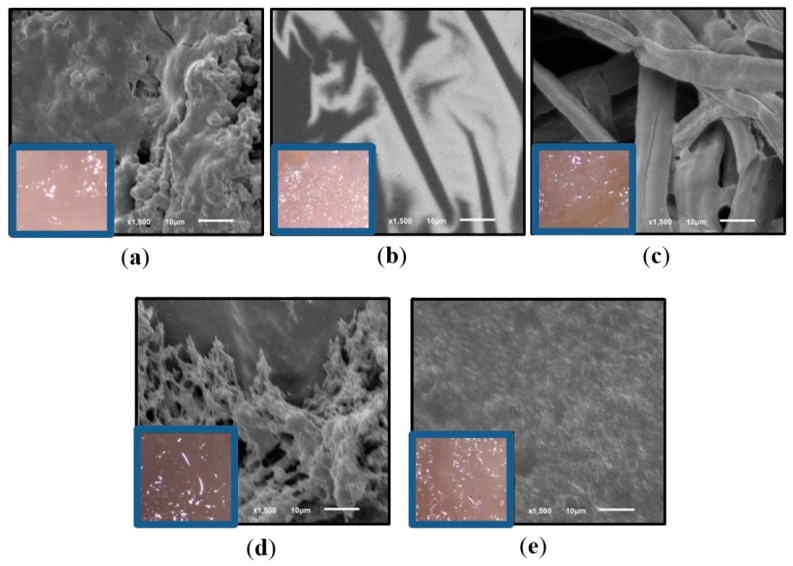
Cellulose pulp treated after washing SEM images (1500×) and optical images (35×—blue squares) for pulp samples treated with the corresponding IL: (**a**) [bmim][Ac]; (**b**) [bmim][Cl]; (**c**) [bmim][HSO_4_]; (**d**) [emim][Ac]; (**e**) [emim][Cl].

The change in color of these pulps can be attributed to several phenomena: first, the ILs in the presence of some residual ions from the synthesis or condensed gas or water vapor might result in color changes [57,58,59]; additionally the possible partial hydrolysis of cellulose fibers into sugars can results in different molecular species as a result of the subsequent breakdown of sugars by oxidative reactions that give the characteristic brown color [60,61]. To confirm whether or not the fibers were degraded or hydrolyzed as a first step, optical and SEM images were taken from these samples (corresponding to Sample 1 shown in Figure 1). These images are presented in Figure 5. 

The samples presented in Figure 5 exhibit visual differences in texture and color in microscope images (Figure 5 blue squares). The differences were confirmed later with SEM images at 1500× (see Figure 5a–e). In the first place, we notice a complete plasticized cellulose after treatment with the ILs derived from acetate anion (see Figure 5a,d)—[bmim][Ac] and [emim][Ac] correspondingly. The plasticized structures were similar to a smooth gel, whiter in the case of [bmim][Ac] in contrast to a less white structure in the case of [emim][Ac], attributed to the presence of residual IL rather than the presence of hydrolyzed fractions.

On the other hand, for IL [bmim][Cl], the regenerated pulp had a similar appearance than hard rubber, evidently due to a strong interaction between the water, cellulose and [bmim][Cl], forming a very stable gel where it was not possible to confirm the presence of fibers by SEM. Finally, for ILs [bmim][HSO_4_] and [emim][Cl], darker pulp samples were regenerated indicating hydrolysis. The SEM images reveal a refined pulp fiber for [emim][Cl] (see Figure 5e), but in the case of [bmim][HSO_4_], this refined pulp was accompanied for almost integral cellulose fibers (see Figure 5c). 

Previous works have shown that the disruption of hydrogen bonds inside cellulose is a critical factor in the dissolution process. The structural features of cellulose hydroxyl groups facilitate active intermolecular hydrogen bonding [62]. ILs containing chloride [Cl] and acetate [OAc] anions have been more widely used for cellulose dissolution [63]. ILs with chloride anion solubilize cellulose through the establishment of hydrogen bonds; meanwhile, for ILs containing acetate, the bonding is accompanied by the simultaneous conjugation of cellulosic reducing ends [22,45,62,63,64]; this explains why these ILs are better for the dissolution of cellulose compared to [bmim][HSO_4_]. 

#### 3.1.2. Molecular Dynamics Results

NPT simulations were carried out to predict the liquid density of pure [bmim][Cl] over the temperature range 298.15 K < T < 363.15 K. Simulations were conducted for 80 ns for each desired temperature. Figure S1 (see supporting information) presents the typical evolution of the simulation box total volume (black line) and total energy (blue line), as observed during the simulations. As shown in Figure S1, the system reaches a state of equilibrium in its total energy—about 10 ns of simulation with the total volume being equilibrated after 50 ns. Therefore, we can assume that the reported simulation time of 160 ns reproduces equilibrium density for the IL. Density was calculated (see Table 3) from the average volume after reaching a state of equilibrium. The simulations were performed at specific temperature and water concentration conditions in order to understand the fundamental interactions of ILs + cellulose + water (that might be present by condensation). Additionally IL densities were calculated considering the literature experimental available data [65], with the intention to confirm the potential of the coarse-grained force field to predict macroscopic properties.

Density calculated by MD overpredicts the experimental data available [65] with an average percentage deviation of 7.5%. 

**Figure 6:**
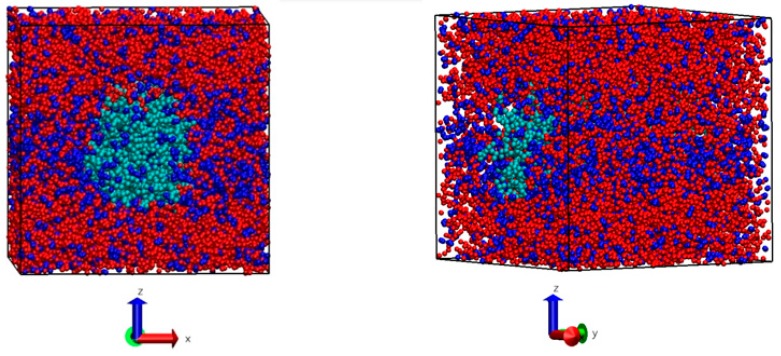
Cellulose fiber (green) solvated with IL (red) and water (blue).

The initial cellulose fiber configuration formed by 36 linear chains, formed in turn by 10 cellobiose units each, was run for one nanosecond relaxation time. The simulation started from *Iα* cellulose polymorph; after the relaxation time, the IL and water molecules were added. Figure 6 shows that the cellulose fibers are at the center of the simulation box (green) solvated with 2826 molecules of IL (red) + 3809 molecules of water (blue), giving a mixture with 10% *w*/*w* water and 18% *w*/*w* cellulose in IL. 

**Figure 7:**
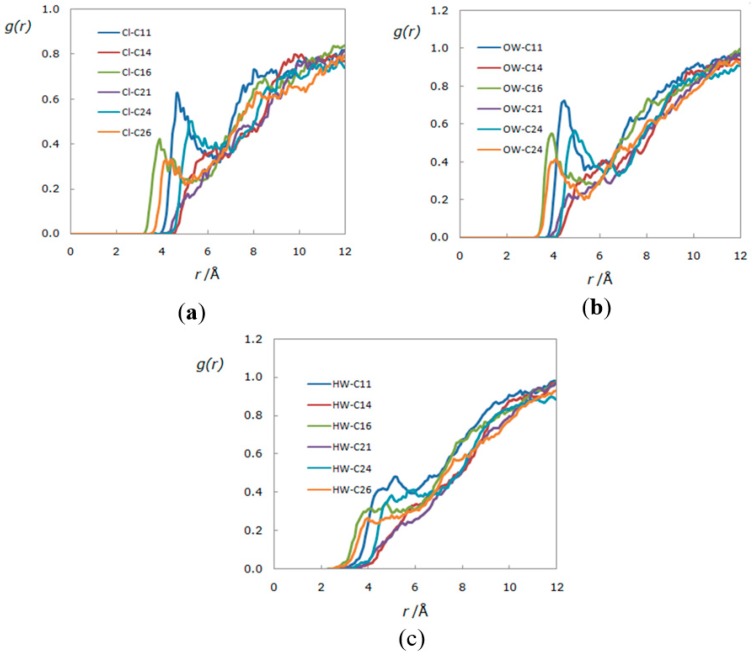
Radial distribution functions of the species at 283.15 K: (**a**) chloride (Cl)–cellulose sites (Cii); (**b**) water oxygen site (OW)–cellulose sites (Cii); (**c**) water hydrogen (HW)–cellulose sites (Cii).

From Figure 6, it is possible to perceive that water and IL molecules compete with each other to interact with the cellulose molecules surrounding the cellulose fibers. This interaction can be seen by the radial distribution functions between the different molecular sites of cellulose, water, and IL sites (Figure 7). 

The radial distribution functions *g(r)* reveal a strong correlation between IL anion and cellulose sites (Figure 7a) as well as between water species and cellulose (Figure 7b,c). This radial distribution shows a competence between IL anion and water species to establish Van der Waals type interactions with cellulose fibers; particularly strong correlations between the IL anion site with the cellubiose sites *C16* and *C26* were found. These strong correlations can be explained considering that such sites contain both carbon number six (carbon belonging to the alquil chain united to glucopyranose ring in the cellulose structure), and this carbon, in particular, posseses the most reactive hydroxyl group [66]. In contrast, sites *C11* and *C24* presented a lower correlation; this could be explained taking into account that both sites include less reactive hydroxyl groups. The other groups with no hydroxyl groups exhibited almost negligible correlation, therefore confirming once more that the critical interaction in the dissolution process is the hydrogen bond type interaction. These results correlate with the experiments, whereafter the addition of water to cellulose + [bmim][Cl] mixtures produced stable hydrogels. However, previous studies report that ILs tolerate water content around 7% [43,44] and theoretically [45] up to 20% *w*/*w* without losing the ability to dissolve cellulose.

### 3.2. Hydrolysis Process

**Figure 8:**
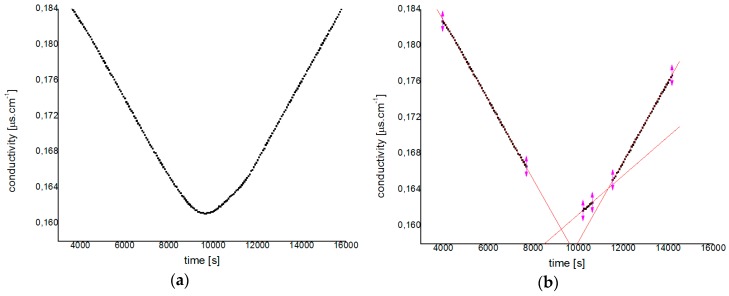
Conductometric titration for cellulose treated pulp with [bmim][HSO_4_]: (**a**) conductivity data; (**b**) identification of three phases for titration.

As discussed previously, the appearance of a refined and dark regenerated cellulose suggests the production of hydrolyzed fractions. Previous examination of samples by AFM confirm the presence of hydrolyzed fractions in samples treated with ILs [bmim][HSO_4_] and [emim][Cl]. These results are in agreement with previously reported findings [24,25,26,29,67,68]. Samples containing hydrolyzed fractions were analyzed after a second washing procedure, as indicated in Figure 1 (Sample 2). The stability of the washed samples was checked by Φ_z_ and ζ measurements. For instance, Figure 8 shows a typical potentiometric titration for [bmim][HSO_4_] treated pulp sample. 

In Figure 8, the conductivity data for the titration of a 0.5% *w*/*w* pulp sample treated with IL [bmim][HSO_4_] is presented. Figure 8a presents the raw data and Figure 8b presents the three phases in the titration process. Phase 1 (linear portion in the left of Figure 8b) represents the phase where the conductivity of the solution decreases when the strong acidic groups are neutralized with NaOH. Phase 2 is equivalent to the neutralization of carboxylic groups, where conductivity reaches a steady state characterized by the decrease of the slope in the conductivity versus time. In Phase 3, the accumulation of base leads to an increase in conductivity, causing once more a drastic change in conductivity slope. The charge is reported as the total acidic group content per dry weight pulp, calculated from the volume of sodium hydroxide solution consumed at the 2nd intersection point [69]. The final charge value was obtained by subtracting this value from the charge obtained in a milliQ^®^ water blank sample. The same procedure was followed for all samples, and the results are summarised in Table 4, together with cellulose nanocrystal (CNC) size. CNC yields are not reported, since it was not possible to completely remove secondary products and unreacted cellulose from the mixture. 

**Figure 9:**
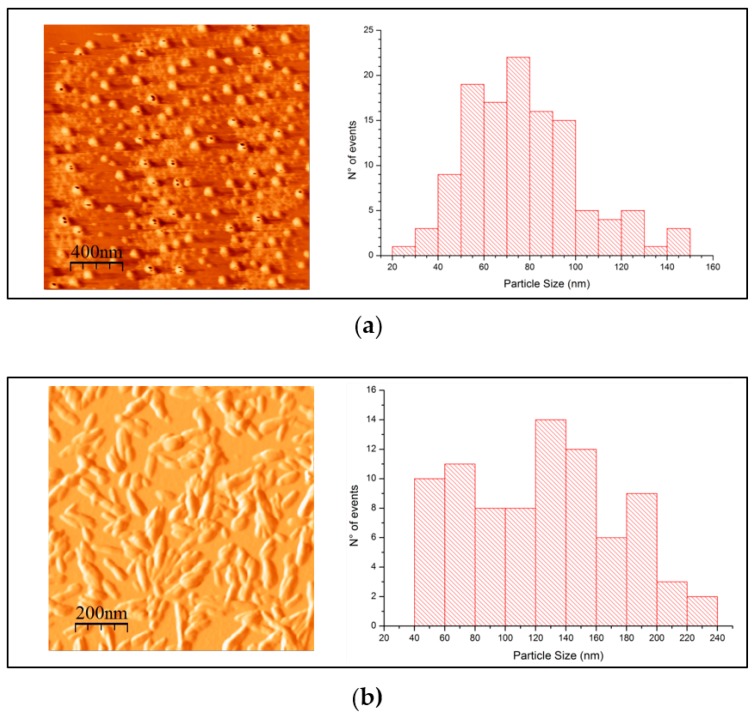
Atomic Force Microscopy images and particle size histogram for IL-treated pulp samples: (**a**) treated pulp with [emim][Cl]; (**b**) treated pulp with [bmim][HSO_4_].

In Table 4, *D* corresponds to the average particle size measured from the AFM images; Φ*_z_* is the zeta potential for samples with 0.05% *w*/*w* solid content at pH= 6.0 and 25 °C, and ζ is the particle charge measured by the SCAN-CM 65:02 conductometric titration method. The values in parentheses correspond to the respective standard deviations. The CNC found in samples treated with IL [bmim][HSO_4_] and [emim][Cl] were substantially different in charge, as shown in Table 4. The CNC synthesized with IL [bmim][HSO_4_] has a higher charge and higher values for z potential; thus, its tendency to agglomerate and precipitate is lower than in the case of particles obtained from [emim][Cl] treatment. This phenomenon can be seen in Figure 9a,b, where AFM images are presented for the resulting treatments.

Despite their charge and potential, the nanoparticles obtained with IL [emim][Cl] were spherical in shape and about 40% smaller than the corresponding nanoparticles synthesized with [bmim][HSO_4_]. Yun tan et al. [54] reported very similar CNC particles with a rod-like shape, diameter of about 15–20 nm, and length of 75–80 nm at 90 °C treatment with [bmim][HSO_4_]. On the other hand, Abushammala et al. [24] reported smaller CNC nanoparticles using IL [emim][Ac] with width ranging from 2 nm to 5 nm and length ranging from 75 nm to 125 nm; in our study, these CNCs were not found for this particular IL. 

From Figure 9, we can confirm that the electrical environment of the CNC particles synthesized has a direct influence on morphology, which means that the unstable CNC suspensions cause more agglomeration, making the visualization of individual nanocrystals challenging. 

Depending on the type of anion and cation of the IL, they possess a higher capacity to dissolve and hydrolyze cellulose fibers. For instance, those that possess anions with a high capacity to accept [H^+^] in their valence bands, in addition to a non-hydrophobic cation or containing long-chain alkyl substituents, possess higher capacity for the dissolution of cellulose [26,70]. In this context, IL [emim][Cl] might be better for dissolution. However, in the hydrolysis process, the chloride anion causes instability to crystals; thus, proper separation of crystals become challenging, requiring more energy compared to the case of [bmim][HSO_4_]. This behavior is analogous to CNC production from the traditional homologous inorganic acids, sulfuric acid [71] and hydrochloric acid [72]. In the particular case of hydrochloric acid, the nanocrystals synthesized possess zero charge and have a high tendency to agglomerate. Therefore, CNC synthesis involving the chloride anion is still challenging from this point of view.

## 4. Conclusions

The ILs studied here were able to dissolve the Chilean Eucalyptus cellulose pulp. In the particular case of ILs [bmim][HSO_4_] and [emim][Cl], CNC crystals were found in the regenerated pulp. Although the nanocrystals were not isolated mainly due to their strong tendency to aggregate, it was possible to describe from the experimental and molecular point of view the processes of dissolution, hydrolysis, and agglomeration of cellulose nanoparticles. It is important to point out that the CNC presence only confirms that the hydrolysis reactions were taking place during the dissolution process. This fact can be attributed to condensed water and possible acid impurities resulting from the ILs synthesis; however, specialized analytical techniques such as HPLC are required in future works, to elucidate the possible molecular species and reaction paths from the hydrolysis or degradation of cellulose.

According to MD results, the main conclusion of this work is that the mesoscopic dynamical behavior of mixtures composed by [bmim][Cl] + cellulose + water can be correctly modeled using the proposed CG model. The force field implemented can predict the atmospheric density of [bmim][Cl] and reproduce the dissolution process of cellulose in IL + water(10% *w*/*w*), showing the high correlation between anion cellulose and water cellulose, mainly due to interactions between hydrogen bonds that finally correlate to the experimental hydrogel formation. Although previous results suggest that better force field parameterization is needed to capture accurate and detailed thermodynamics properties such as surface tensions and diffusion coefficients, this model is an essential first step to understand the IL + cellulose molecular interactions and can help in the construction of other models that use different ILs. This work contributes to understanding the potential of ILs to produce regenerated or hydrolyzed cellulose-derived products. Ultimately, this work offers hints to Chilean forestry industries about the new opportunities to innovate and produce cellulose-based products with minor environmental impact.

## Figures and Tables

**Table 1:** Chemical composition of Bleached Hardwood Kraft Pulp.

Component (%)	Value
Glucan	79.3 ± 0.8
Xylan	14.7 ± 0.6
Total Lignin	0.1 ± 0.1
Ash	0.2 ± 0.1

**Table 2:** Ionic Liquid [bmim][Cl] Mie potential parameters.

Site	ε_0_ (kcal/mol)	N	m	r_o_ (Å)
I1 – I1	0.375	9	6	4.693
I2 – I2	0.345	9	6	4.693
I3 – I3	0.199	9	6	4.120
CT – CT	0.469	9	6	5.249
CI – CI	0.148	12	6	4.232

**Table 3:** Atmospheric liquid densities [bmim][Cl] g^.^cm^−3^.

Temperature	^1^ MD(g^.^cm^−3^)	^2^ EXP(g.cm-3)	^3^ MABD
298.15	1.169	1.082	7.49
323.15	1.157	1.067	7.77
348.15	1.138	1.053	7.47
363.15	1.130	1.045	7.51

^1.^Molecular dynamic simulations results; ^2.^Experimental values reported [65]; ^3.^MABD: Mean absolute percentage deviation.

**Table 4:** CNC obtained from ILs treatment.

IL sample	D(nm)	Φ_z_ (mV)	ζ(µmol_COOH_/g_pulp_)
[bmim][HSO_4_]	123 (48)	−24 (2.5)	0.085 (0.02)
[emim][Cl]	77 (25)	−12 (6)	0 (0)

## Supplementary Materials

The following are available online at https://www.mdpi.com/2073-4360/11/4/673/s1, Figure S1: Total energy (U) (blue line) and total volume (V) (black line) as a function of time (t) in NPT simulation at 298.15 K.
