# Supplementary material for: Dissolution and Hydrolysis of Bleached Kraft Pulp Using Ionic Liquids
Source: Polymers (Basel). 2019 Apr 12;11(4):673. doi: 10.3390/polym11040673 (PMC6523854; doi:10.3390/polym11040673)
Supplement: Supplementary file 1 [file polymers-11-00673-s001.pdf]

Supplementary Information

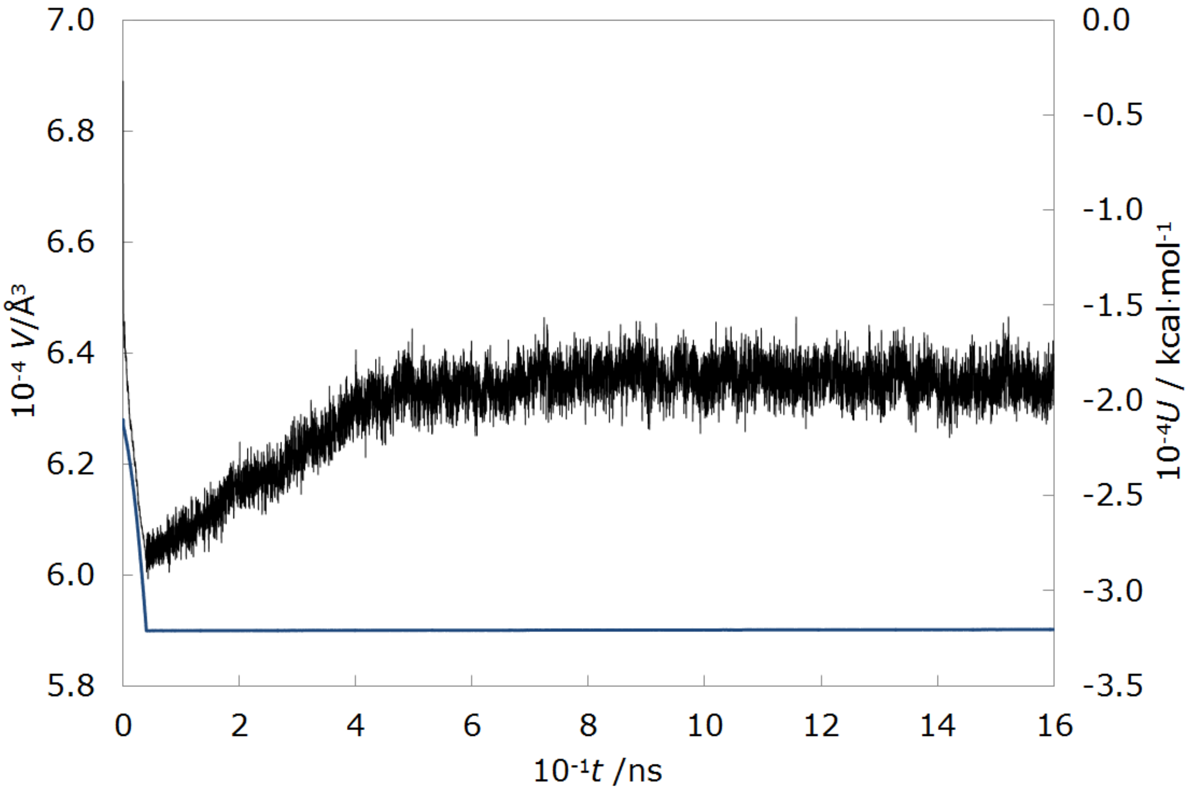

Figure. S1. Total energy (U) (blue line) and total volume (V) (black line) as a function of time (t) in NPT simulation at 298.15 K.
